# Influenza Activity — United States, 2012–13 Season and Composition of the 2013–14 Influenza Vaccine

**Published:** 2013-06-14

**Authors:** World Health Organization Collaborating Center for Surveillance, Epidemiology, and Control of Influenza, Lynnette Brammer, Krista Kniss, Scott Epperson, Lenee Blanton, Desiree Mustaquim, Craig Steffens, Tiffany D’Mello, Alejandro Perez, Rosaline Dhara, Sandra S. Chaves, Anwar Abd Elal, Larisa Gubareva, Teresa Wallis, Xiyan Xu, Julie Villanueva, Joseph Bresee, Nancy Cox, Lyn Finelli, Fiona Havers

**Affiliations:** Influenza Div, National Center for Immunization and Respiratory Diseases, CDC; EIS Officer

During the 2012–13 influenza season in the United States, influenza activity* increased through November and December before peaking in late December. Influenza A (H3N2) viruses predominated overall, but influenza B viruses and, to a lesser extent, influenza A (H1N1)pdm09 (pH1N1) viruses also were reported in the United States. This influenza season was moderately severe, with a higher percentage of outpatient visits for influenza-like illness (ILI), higher rates of hospitalization, and more reported deaths attributed to pneumonia and influenza compared with recent years. This report summarizes influenza activity in the United States during the 2012–13 influenza season (September 30, 2012–May 18, 2013) as of June 7, 2013, and reports the recommendations for the components of the 2013–14 Northern Hemisphere influenza vaccine.

## Viral Surveillance

**FIGURE 1:**
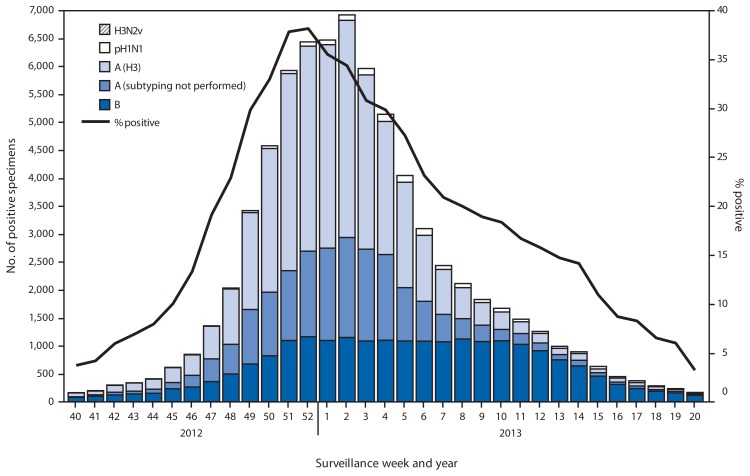
Number and percentage of respiratory specimens testing positive for influenza reported to CDC, by type and surveillance week and year — World Health Organization and National Respiratory and Enteric Virus Surveillance System collaborating laboratories, United States, September 30, 2012–May 18, 2013

During September 30, 2012–May 18, 2013, World Health Organization and National Respiratory and Enteric Virus Surveillance System collaborating laboratories in the United States tested 311,333 specimens for influenza viruses; 73,130 (23%) were positive (Figure 1). Of the positive specimens, 51,675 (71%) were influenza A viruses, and 21,455 (29%) were influenza B viruses. Among the seasonal influenza A viruses, 34,922 (68%) were subtyped; 33,423 (96%) were influenza A (H3N2) viruses, and 1,497 (4%) were pH1N1 viruses. In addition, two variant influenza A (H3N2v) viruses were identified.†

Typically the influenza season is said to begin when certain key indicators remain elevated for a number of consecutive weeks. One of these indicators is the percent of respiratory specimens testing positive for influenza. The proportion of specimens testing positive for influenza during the 2012–13 season first exceeded 10% during the week ending November 10, 2012 (week 45), and peaked at 38% during the week ending December 29, 2012 (week 52).

Since the start of the 2012–13 season, influenza A (H3N2) viruses have predominated nationally, followed by influenza B viruses; pH1N1 viruses have been identified less frequently. The relative proportion of each type and subtype varied by geographic U.S. Department of Health and Human Services region§ and week. Influenza A viruses predominated until the end of February, with influenza B viruses predominating from the week ending February 23, 2013 (week 8) through the week ending May 18, 2013 (week 20).

Regional differences were observed in the timing of influenza activity and the relative proportions of circulating viruses. Using the percentage of specimens testing positive for influenza to determine the peak of influenza activity, Region 4 activity peaked earliest, during the week ending December 8, 2012 (week 49), and Region 9 activity peaked latest, during the week ending January 26, 2013 (week 4). The highest proportion of influenza B viruses was observed in Region 6 (42%) and the lowest proportion of influenza B viruses was detected in Region 1 (15%).

## Novel Influenza A Viruses

During the 2012–13 influenza season, one case of human infection with a variant influenza A (H3N2) (H3N2v) virus was reported in each of two states, Minnesota and Iowa. Both infections occurred in children, one with known exposure to swine. Both patients recovered fully.

## Antigenic Characterization

CDC has antigenically characterized 2,452 influenza viruses collected since October 1, 2012, and submitted by U.S. laboratories, including 252 pH1N1 viruses, 1,324 influenza A (H3N2) viruses, and 876 influenza B viruses. Of the 252 pH1N1 viruses tested, 249 (98.8%) were characterized as A/California/7/2009-like, the influenza A(H1N1) component of the 2012–13 influenza vaccine. Three viruses (1.2%) of the 252 tested showed reduced titers with ferret antiserum raised against A/California/7/2009. Of the 1,324 influenza A (H3N2) viruses, 1,319 (99.6%) were antigenically similar to the cell-propagated A/Victoria/361/2011 reference virus; most viruses tested were cell-propagated. The H3N2 vaccine component for the 2012–13 Northern Hemisphere season was egg-propagated A/Victoria/361/2011; the use of egg-propagated vaccine viruses is a current regulatory requirement for vaccine production. Five (0.4%) of the 1,324 tested showed reduced titers with antiserum produced against cell-propagated A/Victoria/361/2011.

Of the 876 influenza B viruses tested, 581 (66.3%) belonged to the B/Yamagata lineage, and were characterized as B/Wisconsin/1/2010-like, the influenza B component for the 2012–13 Northern Hemisphere influenza vaccine. A total of 295 (33.7%) viruses tested belonged to the B/Victoria lineage.

## Resistance to Antiviral Medications

Since October 1, 2012, a total of 3,626 influenza virus specimens have been tested for antiviral resistance. All 961 influenza B viruses tested were sensitive to both oseltamivir and zanamivir. Among 2,123 influenza A (H3N2) viruses tested, one (0.05%) was found to be resistant to oseltamivir alone and one (0.05%) to both oseltamavir and zanamivir. Among the 542 pH1N1 viruses tested for resistance to oseltamivir, two (0.4%) were resistant, and all of the 258 viruses tested for resistance to zanamivir were sensitive. High levels of resistance to the adamantanes (amantadine and rimantadine) persist among influenza A viruses currently circulating globally (the adamantanes are not effective against influenza B viruses).

## Composition of the 2013–14 Influenza Vaccine

The Food and Drug Administration’s Vaccines and Related Biological Products Advisory Committee has recommended that the 2013–14 influenza trivalent vaccines used in the United States contain an A/California/7/2009(H1N1)pdm09-like virus, an A(H3N2) virus antigenically like the cell-propagated A/Victoria/361/2011 virus (A/Texas/50/2012), and a B/Massachusetts/2/2012-like (B/Yamagata lineage) virus. A/Texas/50/2012 is an egg-propagated A(H3N2) virus antigenically similar to cell-propagated A/Victoria/361/2011. The committee recommended that A/Texas/50/2012 be used as the H3N2 vaccine component because of antigenic changes in A/Victoria/361/2011 vaccine virus resulting from mutations acquired during growth in eggs. The committee also recommended that quadrivalent vaccines contain a B/Brisbane/60/2008-like (B/Victoria lineage) virus (1). These recommendations were based on global influenza virus surveillance data related to epidemiology, antigenic and genetic characteristics, and serological responses to 2012–13 seasonal vaccines, and the availability of candidate strains and reagents.

## Outpatient Illness Surveillance

**FIGURE 2:**
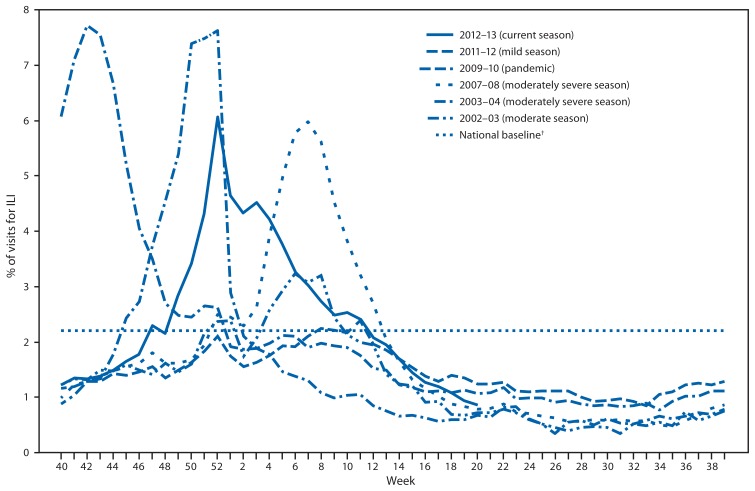
Percentage of visits for influenza-like illness (ILI)^*^ reported to CDC, by surveillance week and year — U.S. Outpatient Influenza-Like Illness Surveillance Network, United States, September 30, 2012–May 18, 2013, and selected previous seasons ^*^ Defined as a temperature of ≥100.0°F (≥37.8°C), oral or equivalent, and cough or sore throat, in the absence of a known cause other than influenza. ^†^The national baseline is the mean percentage of visits for ILI during noninfluenza weeks for the previous three seasons plus two standard deviations. A noninfluenza week is defined as periods of two or more consecutive weeks in which each week accounted for <2% of the season’s total number of specimens that tested positive for influenza. Use of the national baseline for regional data is not appropriate.

Nationally, the weekly percentage of outpatient visits for ILI¶ to health-care providers participating in the U.S. Outpatient Influenza-Like Illness Surveillance Network (ILINet) exceeded the national baseline level of 2.2% for 15 weeks during the 2012–13 influenza season (Figure 2). The peak percentage of outpatient visits for ILI was 6.1%, and occurred in the week ending December 29, 2012 (week 52). In contrast, the peak percentage of outpatient visits for ILI during the previous influenza season (2011–12) was 2.4% and occurred in mid-March. During the 2007–08 and 2010–11 influenza seasons, both of which had influenza A (H3N2) virus as the predominant circulating virus, the peak percentage of outpatient visits for ILI was 6.0% and 4.6%, respectively; both peaks occurred in mid-February. During the 2012–13 season, on a regional level, the percentage of visits for ILI exceeded region-specific baselines in all 10 regions. ILINet data are used to produce a weekly state-level measure of ILI activity varying from minimal to high: the number of states experiencing high ILI activity peaked during the week ending December 29, 2012 (week 52) with 35 states.

## State-Specific Activity Levels

State and territorial epidemiologists report the geographic distribution of influenza in their states through a weekly influenza activity code. The geographic distribution of influenza activity was most extensive during the week ending January 12, 2013 (week 2), when 48 states reported widespread influenza activity and two states reported regional influenza activity. The week ending May 18, 2013 (week 20) was the first week no state or territory reported regional or widespread influenza activity. The number of states reporting widespread or regional activity during the peak week of activity has ranged from 20 to 50 states during the previous four influenza seasons (Influenza Division, CDC, unpublished data, 2013).

## Influenza-Associated Hospitalization

**FIGURE 3:**
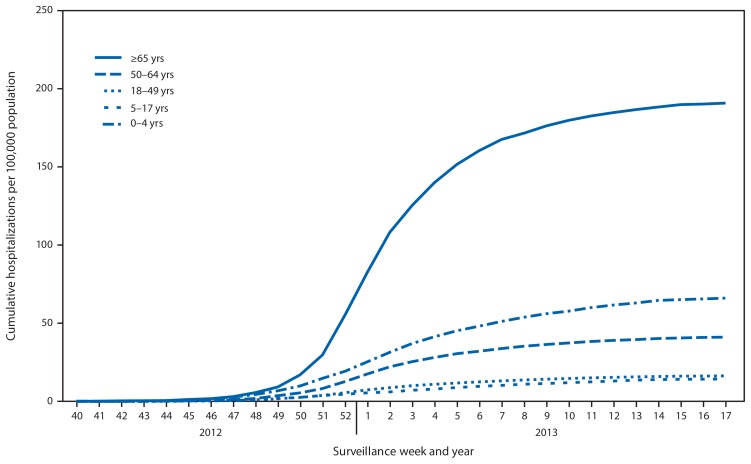
Cumulative hospitalization rates for laboratory-confirmed influenza, by age group and surveillance week and year — FluSurv-NET^*^ surveillance system, United States, October 1, 2012–April 30, 2013

CDC monitors hospitalizations associated with laboratory-confirmed influenza virus infections using the FluSurv-NET** surveillance system. Cumulative hospitalization rates (per 100,000 population) were calculated by age group based on 12,337 total hospitalizations resulting from influenza during October 1, 2012–April 30, 2013. Among 12,293 cases with influenza type specified, 9,767 (79.2%) were associated with influenza A and 2,492 (20.2%) with influenza B; and 34 (0.3%) were associated with influenza A and influenza B co-infections; 44 (0.4%) had no virus type information available. Persons aged ≥65 years accounted for approximately 50% of reported cases. The cumulative incidence†† for all age groups since October 1, 2012, was 44.3 per 100,000 (Figure 3). The cumulative hospitalization rate (per 100,000 population) by age group for this period was 66.2 (0–4 years), 14.5 (5–17 years), 16.4 (18–49 years), 41.2 (50–64 years), and 191.2 (≥65 years). During the past four influenza seasons, age-specific hospitalization rates ranged from 15.8 to 72.8 (0–4 years), 4.0 to 27.3 (5–17 years), 3.6 to 23.1 (18–49 years), 5.1 to 30.8 (50–64 years), and 13.5 to 65.9 (≥65 years).

What is already known on this topic?

CDC collects, compiles, and analyzes data on influenza activity year-round in the United States. The influenza season generally begins in the fall and continues through the winter and spring months; however, the timing and severity of influenza activity varies by geographic location and season.

What is added by this report?

During the 2012–13 influenza season, influenza A (H3N2), influenza A (H1N1)pdm09, and influenza B viruses cocirculated. In addition, two cases of infection with variant influenza A viruses were reported in the United States. Compared with recent influenza seasons, this season had a higher percentage of outpatient visits for influenza-like illness, higher rates of hospitalizations, and more deaths attributed to pneumonia and influenza.

What are the implications for public health practice?

All unvaccinated persons aged ≥6 months should be offered influenza vaccine throughout the influenza season. In addition, timely empiric antiviral treatment is recommended for patients with severe, complicated, or progressive influenza illness; those at higher risk for influenza complications; or those for whom treatment can be started within 48 hours of illness onset. In addition, influenza surveillance, including for novel influenza viruses, should continue through the summer months, and physicians should consider influenza as a cause of respiratory illness outside of the typical season.

As of June 1, 2013, among the FluSurv-NET adult patients for whom medical chart data were available, the most frequent underlying conditions were chronic lung disease (27%), cardiovascular disease (45%), and metabolic disorders (39%). Among children hospitalized with laboratory-confirmed influenza and for whom medical chart data were available, 46% did not have any recorded underlying conditions, and 22% had underlying asthma or reactive airway disease. Among the 819 hospitalized women of childbearing age (15–44 years), 233 (28%) were pregnant.

## Pneumonia- and Influenza-Related Mortality

**FIGURE 4:**
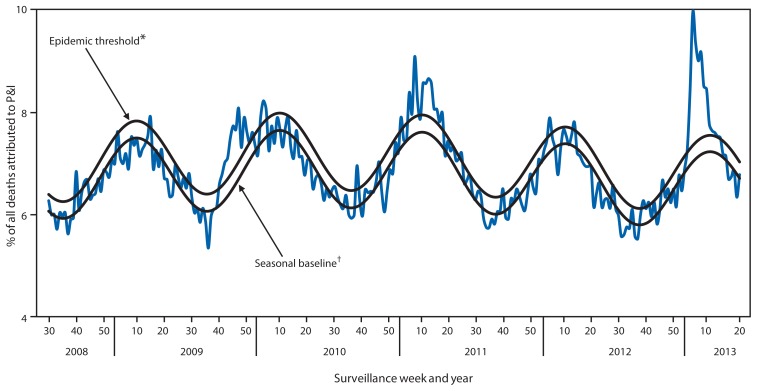
Percentage of all deaths attributable to pneumonia and influenza (P&I), by surveillance week and year — 122 Cities Mortality Reporting System, United States, 2008–May 18, 2013 ^*^ The epidemic threshold is 1.645 standard deviations above the seasonal baseline. ^†^ The seasonal baseline is projected using a robust regression procedure that applies a periodic regression model to the observed percentage of deaths from P&I during the preceding 5 years.

During the 2012–13 influenza season, the percentage of deaths attributed to pneumonia and influenza (P&I) exceeded the epidemic threshold for 13 consecutive weeks spanning December 30, 2012 to March 30, 2013 (weeks 1–13). The percentage of deaths attributed to P&I peaked at 9.9% during the week ending January 19, 2013 (week 3) (Figure 4). From the 2008–09 season through the 2011–12 season, the peak percentage of P&I deaths ranged from 7.9% to 9.1%, and the total number of consecutive weeks at or above the epidemic threshold ranged from 1 to 13 (Influenza Division, CDC, unpublished data, 2013).

## Influenza-Related Pediatric Mortality

For the 2012–13 influenza season, 149 laboratory-confirmed, influenza-associated pediatric deaths were reported. These deaths were reported from 38 states. The states with the greatest numbers of deaths were Texas (18), New York (14), and Florida (eight). The deaths included 11 children aged <6 months, 20 aged 6–23 months, 20 aged 2–4 years, 52 aged 5–11 years, and 46 aged 12–17 years; mean and median ages were 8.2 years and 8.1 years, respectively. Among the 149 deaths, 79 were associated with influenza B viruses, 32 with influenza A (H3) viruses, four with pH1N1 viruses, 31 with an influenza A virus for which the subtype was not determined, one with an influenza virus for which the type was not determined, and two with both an influenza B and influenza A virus.

Since influenza-associated pediatric mortality became a nationally notifiable condition in 2004, the total number of influenza-associated pediatric deaths has previously ranged from 34 to 123 per season; this excludes the 2009 pandemic, when 348 pediatric deaths were reported to CDC during April 15, 2009, through October 2, 2010.

### Editorial Note

The 2012–13 influenza season peaked early and was a moderately severe season, with influenza A (H3N2) viruses predominating. Activity peaked in late December, and influenza A (H3N2) viruses were most commonly reported through the week ending February 16, 2013 (week 7). From the week ending February 23, 2013 (week 8), through the end of the season, influenza B viruses were more commonly reported. The majority of all influenza viruses in specimens sent to CDC for further antigenic characterization were similar to the components of the 2012–13 Northern Hemisphere vaccine.

The peak percentage of outpatient visits for ILI (6.1%) was one of the highest reported since the system began in its current format in 1997. For comparison, the peak percentage of visits for ILI during those 15 seasons ranged from 2.4% for the 2011–12 season to 7.7% during the 2009 H1N1 pandemic. The number and rate of influenza-associated hospitalizations among adults aged ≥65 years during the 2012–13 influenza season are the highest since systematic data collection on laboratory-confirmed, influenza-associated hospitalization in adults began in the 2005–06 season. Hospitalization rates for those aged ≥65 years were 191 per 100,000 population, two and a half times the highest rate previously reported for this age group. With the exception of the 2009 H1N1 pandemic, the number of influenza-associated pediatric deaths reported to CDC for the 2012–13 season was the highest reported since data collection began in 2004. Reported P&I mortality exceeded the epidemic threshold for 13 consecutive weeks. Based on the percentage of specimens testing positive for influenza, the peak of influenza activity for the 2012–13 season, occurring during the week ending December 29, 2012 (week 52), was similar to the 2003–04 season, which peaked during the week ending November 30, 2003 (week 48), and was the earliest since the 2009 H1N1 pandemic, when activity peaked during the week ending October 24, 2009 (week 42).

On March 31, 2013, Chinese health authorities reported a novel avian influenza A (H7N9) virus causing human infection. As of June 7, 2013, 132 cases have been confirmed; many of the infected people are reported to have had close contact with poultry. The virus has only been seen in mainland China and Taiwan; no cases have been reported in the United States. Unlike the variant influenza A (H3N2)v virus associated with swine exposure in the United States, which generally caused mild illness, the avian influenza A (H7N9) virus has caused severe illness in the majority of cases in humans, and approximately 27% of identified cases have been fatal (2).

Testing for seasonal influenza viruses and monitoring for novel influenza A virus infections should continue year-round, as should specimen submission to CDC for further antigenic and genetic analysis and antiviral resistance monitoring. A total of 308 infections with variant influenza viruses (304 H3N2v viruses, three H1N2v viruses, and one H1N1v virus) were reported from 10 states during the summer and fall of 2012, before the start of the 2012–13 influenza season, and two cases of H3N2v were detected during the 2012–13 season. The H3N2v virus circulated in pigs in 2010 and was first detected in humans in 2011, when 12 cases were identified. Most of these infections occurred in children with prolonged exposure to pigs at agricultural fairs. Limited human-to-human spread of this virus was detected, but no sustained community spread of H3N2v was identified (3). However, this increase in H3N2v cases in 2012, and the recent emergence of the novel avian influenza A (H7N9) virus in China, further emphasizes the importance of continuing to monitor for novel influenza A viruses.

Although summer influenza activity in the United States typically is low, cases of influenza and even sporadic outbreaks are detected in the United States throughout the summer. Health-care providers should remain vigilant and consider influenza as a potential cause of summer respiratory illnesses. They also should consider novel influenza viruses in persons with ILI and swine exposure, and those with severe acute respiratory infection after travel to China. Public health laboratories should immediately send to CDC virus specimens that they cannot type or subtype using standard methods and submit all specimens that are otherwise unusual, including all summer specimens, as soon as possible after identification.

Since 2010, CDC has recommended annual influenza vaccination for all persons aged ≥6 months, preferably in the fall before the U.S. influenza season begins (4). However, during other times of the year, persons who have not received the vaccine for the current season should be vaccinated before traveling to parts of the world where influenza activity is ongoing. This is particularly important for persons at high risk for influenza-related complications.§§ This recommendation also applies to persons traveling within the temperate regions of the Southern Hemisphere or as part of large tourist groups (e.g., on cruise ships) that might include persons from other parts of the world where influenza activity is ongoing (5). Persons should be vaccinated at least 2 weeks before travel for immunity to develop. Travelers also should be aware that all Northern Hemisphere influenza vaccine manufactured for the 2012–13 season expires by June 30, 2013, after which influenza vaccines will not be available in the United States until the 2013–14 vaccine is available in the fall.

As a supplement to vaccination, influenza antiviral drugs are an important adjunct to reduce the impact of influenza. Based on recommendations of the Advisory Committee on Immunization Practices, antiviral treatment is recommended as soon as possible for patients with confirmed or suspected influenza who have severe, complicated, or progressive illness; who require hospitalization; or who are at higher risk for influenza-related complications (6). Antiviral treatment also may be considered for outpatients with confirmed or suspected influenza who do not have known risk factors for severe illness if treatment can be initiated within 48 hours of illness onset. In addition, if a clinician does suspect that a patient might have an infection caused by a novel influenza virus, prompt empiric antiviral therapy is recommended. Recommended antiviral medications include oseltamivir and zanamivir. Recent viral surveillance and resistance data indicate that the majority of currently circulating influenza viruses are sensitive to these medications. Amantadine and rimantadine should not be used because of sustained high levels of resistance to these drugs among circulating influenza A viruses.

## Footnotes

* Additional information on influenza surveillance and reporting systems in the United States, methods, and levels of activity is available at http://www.cdc.gov/flu/weekly/overview.htm.

† Influenza viruses that normally circulate in pigs are called “variant” viruses when they are found in humans. Influenza A (H3N2) variant viruses (“H3N2v” viruses) with the matrix (M) gene from the 2009 H1N1 pandemic virus were first detected in humans in July 2011. Since then, 319 cases of H3N2v infection have been confirmed in humans, mostly associated with prolonged exposure to pigs at agricultural fairs.

§ Additional information available at http://www.hhs.gov/about/regionmap.html.

¶ Defined as a temperature of ≥100.0°F (≥37.8°C), oral or equivalent, and cough or sore throat, in the absence of a known cause other than influenza.

** FluSurv-NET covers approximately 80 counties in the 10 Emerging Infections Program states (California, Colorado, Connecticut, Georgia, Maryland, Minnesota, New Mexico, New York, Oregon, and Tennessee) and additional Influenza Hospitalization Surveillance Project (IHSP) states. IHSP began during the 2009–10 season to enhance surveillance during the 2009 H1N1 pandemic. IHSP sites included Iowa, Idaho, Michigan, Oklahoma, and South Dakota during 2009–10 season; Idaho, Michigan, Ohio, Oklahoma, Rhode Island, and Utah during the 2010–11 season; Michigan, Ohio, Rhode Island, and Utah during the 2011–12 season; and Iowa, Michigan, Ohio, Rhode Island, and Utah during the 2012–13 season.

†† Incidence rates are calculated using population estimates for the counties included in the surveillance catchment area. Laboratory confirmation is dependent on clinician-ordered influenza testing, and testing for influenza often is underused because of the poor reliability of rapid test results and greater reliance on clinical diagnosis for influenza. As a consequence, cases identified as part of influenza hospitalization surveillance likely are an underestimation of the actual number of persons hospitalized with influenza.

§§ Additional information available at http://www.cdc.gov/flu/about/disease/high_risk.htm.
